# The relationship between perfectionism and career decision-making difficulties in college students: a chain mediation model based on core self-evaluations and psychological resilience

**DOI:** 10.3389/fpsyg.2026.1824328

**Published:** 2026-06-09

**Authors:** Yanqin Tang, Xiaoming Liang, Xing Su

**Affiliations:** 1 School of Marxism, Dongguan Polytechnic, Dongguan, Guangdong, China; 2 College of Economics and Management, Guangxi Vocational University of Agriculture, Nanning, Guangxi, China; 3 School of Marxism, Guangxi Vocational University of Agriculture, Nanning, Guangxi, China

**Keywords:** career decision-making difficulties, college students, core self-evaluations, perfectionism, psychological resilience

## Abstract

**Background:**

In recent years, career decision-making difficulties among college students have drawn increasing attention, significantly relating to their mental health and personal development.

**Method:**

This study surveyed 1,000 college students through a convenience sampling procedure using an online questionnaire. A chain mediation model was developed and validated by using standardized scales to measure perfectionism, core self-evaluation, psychological resilience and career decision-making difficulty.

**Result:**

The findings demonstrated that positive perfectionism is connected to career decision-making difficulties in college students through core self-evaluations and psychological resilience, and the indirect path through core self-evaluations was more prominent than the path through psychological resilience and the chain path through core self-evaluations and psychological resilience. Negative perfectionism also is associated with career decision-making difficulties through core self-evaluations and psychological resilience, with the indirect path through core self-evaluations again showing the strongest statistical association. In addition, the chain path through core self-evaluations and psychological resilience was stronger than the path through psychological resilience alone.

**Conclusion:**

Overall, perfectionism may be associated with career decision-making difficulties in college students, both directly and indirectly, and it could be linked to these difficulties through its associations with core self-evaluations and psychological resilience. These results should be viewed as statistical correlations rather than causal evidence because of the cross-sectional approach.

## 1 Introduction

In the context of the continued expansion of the graduate population, college students face increasing pressure in making career decisions during their graduation stage (Chen et al., 2021). In China, making career decisions has become a crucial developmental challenge for college students due to the growing number of graduates and the competitive youth labor market. Career decision-making difficulties (CDMD) refer to the difficulties individuals experience before, during, or after making career choices, including lack of readiness, lack of information, and inconsistent information (Gati et al., 1996). Existing research indicates that more than one-quarter of college students experience significant CDMD, highlighting the practical significance of this issue (Yang et al., 2024). CDMD are not only associated with difficulties in career planning and career-related actions, but are also related to negative emotional states, such as anxiety and depression, as well as lower life satisfaction (Atitsogbe et al., 2024; Parola and Marcionetti, 2022). In order to better comprehend college students’ career development process and provide career help in establishments of higher learning, it is crucial to uncover the psychological elements that underlie the challenges they encounter when making career decisions.

CDMD is not merely a direct reflection of the intensifying employment pressure or the insufficiency of career information. In recent years, relevant research has gradually expanded from external employment environments and career information conditions to students’ cognitive evaluation characteristics and psychological resource status in the career selection process (Dev and Arli, 2025; Mittal, 2021; Zhou A. B. et al., 2024). Within this line of research, perfectionism has gradually become an important psychological characteristic for understanding CDMD among college students. Making a career decision typically involves weighing several options, unclear results, and insufficient information. Therefore, the repeated comparisons, hesitations, and evasions associated with a person’s career choices may be intimately linked to their assessment of the desired outcome, the danger of error, and the performance of the decision-making process. Previous studies have shown that negative perfectionistic worries are typically linked to increased CDMD levels. In contrast, adaptive perfectionism, characterized by a systematic, planned, and structured pursuit of goals, may be associated with more organized career exploration and career planning (Chen et al., 2022; Frost et al., 1990; Kang et al., 2020). At the same time, research on CDMD has gradually paid attention to students’ general psychological resources in contexts of career uncertainty. Among these resources, core self-evaluations and psychological resilience correspond, respectively, to students’ self-belief resources and stress-adaptation resources in career decision-making. The former pertains to students’ overall assessment of their own abilities, values and sense of control. Research already conducted has demonstrated that it is connected to career choice confidence, career clarity, and CDMD (Shen et al., 2021; Tian et al., 2021). The latter involves students’ adaptive state in stressful, frustrating and ambiguous situations and has also been found to be related to career exploration, emotional stability and levels of CDMD (He et al., 2024; Pang et al., 2021). Therefore, core self-evaluations and psychological resilience provide complementary perspectives for understanding CDMD among college students from the dimensions of self-belief and stress adaptation.

Comprehensive studies on the connection between various types of perfectionism and the stress-reduction and self-belief techniques that students employ when faced with career uncertainty, despite the fact that earlier studies have looked at the connection between perfectionism and CDMD among college students. In particular, compared with more established career-specific or contextual explanatory perspectives, such as career adaptability, career decision-making self-efficacy, vocational identity, and social support, core self-evaluations and psychological resilience represent more general intrapersonal psychological resources. These two aspects have usually been examined separately. Whether they can be jointly represented in the same model as self-belief and stress adaptation resources, and whether they present continuous statistical correlations with different perfectionism tendencies and career decision difficulties, still need further examination. Given this, the current study integrates cognitive behavioral theory (CBT) (Beck et al., 2024) and social cognitive career theory (SCCT) (Lent et al., 1994) to investigate the connections between perfectionism, core self-evaluations, psychological resilience, and CDMD among college students. The aim is to construct an integrated psychological resource model at three levels: cognitive evaluation, self-belief, and stress adaptation resources, to complement the explanatory paths of existing research that mainly focus on career-specific variables and external support conditions. The observed links are only understood as theory-driven statistical correlations due to the cross-sectional method employed in this research, which does not show temporal sequences or causal links between the variables.

## 2 Theoretical foundation and research hypotheses

### 2.1 Theoretical foundation

CBT, which combines behavioral and cognitive theories, emphasizes how emotions, behavior, and cognition interact dynamically. It highlights that interpretations of situations, rather than situations themselves, trigger emotional responses and subsequent behaviors (Beck et al., 2024). CBT focuses on examining how maladaptive cognitive patterns, such as cognitive distortions and irrational beliefs, are closely linked to the persistence of emotional distress and behavioral difficulties (e.g., avoidance, procrastination) (Rozental, 2020). In this research, negative perfectionism is viewed as a maladaptive cognitive tendency. Its core cognitive features, such as rigid standards, fear of mistakes, and self-critical evaluation, provide a basis for understanding decision-related tension and hesitation from a cognitive perspective when facing career choices. Within the Chinese Frost Multidimensional Perfectionism Scale (FMPS) framework used in this study, positive perfectionism is reflected by organization, which emphasizes orderliness, planning, and structured goal pursuit. Rigid standards, fear of making mistakes, and self-critical appraisal are more strongly associated with negative perfectionism, which manifests as parental demands, personal standards, anxiety about mistakes, and concerns about actions. From a CBT perspective, these two forms of perfectionism can be understood as different cognitive-evaluative tendencies in career decision-making contexts. Therefore, CBT aids in understanding how different perfectionistic tendencies can be theoretically positioned as distinct cognitive styles in career decision-making contexts.

Based on Social Cognitive Theory, SCCT was developed by Lent et al. (1994) and highlights the ways in which career objectives, actions, and outcomes are influenced by both environmental and individual characteristics (such as hobbies, skills, values, self-efficacy, and expectations for results) (Rasdi and Ahrari, 2020). Within this framework, core self-evaluations are positioned as students’ general self-beliefs about competence, worth, and control. These beliefs are relevant to how students evaluate their own capacity when facing career-related tasks. Under the SCCT framework, psychological resilience represents a person’s ability to maintain or restore adaptive functioning in the face of stressors, obstacles, or disappointments while making career decisions. Thus, SCCT provides a theoretical basis for positioning core self-evaluations and psychological resilience as individual psychological resources when choosing a career.

In the present study, CBT and SCCT are used to explain different but connected parts of the proposed model. Specifically, CBT explains the cognitive-evaluative features of perfectionism in career decision-making. Negative perfectionism reflects rigid standards, fear of mistakes, and self-critical evaluation, whereas positive perfectionism, represented by the organization dimension, reflects orderliness, planning, and structured goal pursuit. SCCT explains the role of core self-evaluations and psychological resilience as self-belief and adaptation resources in career decision-making. Thus, CBT clarifies the cognitive characteristics of perfectionism, while SCCT explains why self-belief and resilience-related resources are relevant to CDMD. Together, the two frameworks provide an integrated basis for examining perfectionism, core self-evaluations, psychological resilience, and CDMD in the same model.

### 2.2 Perfectionism and career decision-making difficulties

Excessively high personal standards, strict self-evaluation, and a propensity for extreme, all-or-nothing thought processes are prominent characteristics of perfectionism (Frost et al., 1990). With further research, researchers have identified two categories of perfectionism: negative perfectionism and positive perfectionism (Parker, 1997; Zhang et al., 2021). However, this distinction does not imply simplifying perfectionism into a straightforward positive–negative dichotomy. On the contrary, in existing literature, perfectionism is generally regarded as a multi-dimensional construct, with different dimensions potentially showing distinct patterns of association with career-related outcomes and psychological adaptation (Stoeber and Otto, 2006; Wang et al., 2020). Therefore, the positive–negative classification adopted in this study is an operational distinction based on the dimensional structure of the Chinese version of the Frost Multidimensional Perfectionism Scale, rather than denying the multidimensional nature of perfectionism itself. In studies using the Chinese FMPS, positive perfectionism is commonly linked to the organization dimension, which reflects orderliness, planning, and structured goal pursuit, whereas negative perfectionism is commonly connected to misgivings about acts, personal standards, worries about blunders, and expectations from parents (Hu et al., 2023). Prior research has indicated a robust connection between perfectionism and CDMD (Chen et al., 2022; Kang et al., 2020; Tian and Hou, 2024; Wang et al., 2020), with different types of perfectionism showing different relationship patterns with CDMD among college students. For example, the study by Tian and Hou (2024) found that positive perfectionism is adversely related to CDMD, suggesting a pattern of greater ease in planning and action among those with flexible high standards. In contrast, according to Chen et al. (2022), a positive correlation has been observed between negative perfectionism and Chinese college students’ CDMD. Negative perfectionists, who demand perfection and cannot tolerate flaws, may experience more hesitation, anxiety, and avoidance when making career decisions. The following hypothesis for this study is derived from this analysis:

*H1a*: Positive perfectionism has a negative correlation with CDMD among college students.

*H1b*: Negative perfectionism has a favorable correlation with CDMD among college students.

### 2.3 The mediating role of core self-evaluations

Self-efficacy, self-esteem, emotional stability, and an internal locus of control are examples of core self-evaluations, which are a person’s fundamental judgment of their own abilities and value (Lin et al., 2021; Liu et al., 2025). Prior research has demonstrated that among college students, core self-evaluations are connected to lower CDMD (Kou et al., 2024; Udayar et al., 2020). Greater levels of self-identity and internal control are common personality features among those with greater core self-evaluations. They may show clearer self-understanding and stronger confidence when evaluating career options (Ran et al., 2022; Shen et al., 2021; Zhu et al., 2021). For example, according to Shen et al. (2021), college students who score higher on core self-evaluations are generally more likely to report stronger career calling and clearer career engagement. The development of professional plans, proactive self- and career-exploration, and the clarification of career goals are all associated with this sense of calling, which also relates to fewer CDMD (Tian et al., 2021).

Perfectionism is closely related to core self-evaluations. Perfectionists often have high levels of self-efficacy and may be intrinsically motivated to achieve, which supports a positive outlook (Williams and Edwards, 2022). However, their lofty goals and standards for themselves are frequently unachievable, which may be linked to high levels of self-doubt and decreased core self-evaluations (Fearn et al., 2022; Halsell, 2023). As an example, Fearn et al. (2022) discovered that those who have negative perfectionism typically report lower core self-evaluations by setting unattainable standards, catastrophizing fear of failure, engaging in harsh self-criticism, and adopting avoidance behaviors. In contrast, those with positive perfectionism tend to report higher core self-evaluations by setting realistic high goals, putting in effort, experiencing controllable success, learning from setbacks, and maintaining flexible thinking (Azizi et al., 2017; Zhang et al., 2025). Based on this analysis, this research postulates the following:

*H2a*: Core self-evaluations mediate in part the relationship between positive perfectionism and CDMD.

*H2b*: Core self-evaluations mediate in part the relationship between negative perfectionism and CDMD.

### 2.4 The mediating role of psychological resilience

The ability to adjust, recover, and preserve mental health in the face of stress, hardship, or difficult circumstances is known as psychological resilience (Zeng et al., 2023). Prior studies have discovered a negative relationship between psychological resilience and CDMD (Pang et al., 2021; Zhou W. et al., 2024). As stated by SCCT (Lent et al., 1994), psychological resilience, as an important psychological resource, helps individuals sustain a sense of control and persistence while choosing a career. For example, Pang et al. (2021) surveyed of 666 Chinese college students and found that those who were more psychologically resilient reported fewer difficulties choosing a job.

Perfectionism is a rather consistent personality trait that has been connected to differences in psychological resilience (Doyle and Catling, 2022; Islim and Ahmad, 2025). Islim and Ahmad (2025) stated in a study involving 553 college students that perfectionism explained 31.2% of the variance in psychological resilience. Different types of perfectionism may have different associations with psychological resilience. As an example, people with greater amounts of positive perfectionism are more inclined to regulate their goals through self-reflection, which is associated with greater psychological resilience. In contrast, because of their unattainable ideals, people with higher levels of negative perfectionism frequently experience higher levels of stress, which is linked to lower resilience (Chen et al., 2024; Wan et al., 2023). With ongoing research, some scholars have investigated psychological resilience as a mediating factor, exploring its role in the indirect pathway linking difficulties in making career choices. However, it is yet unclear if psychological resilience acts as a mediator in the relationship between perfectionism and CDMD. In order to close this gap, the current study investigates potential mechanisms through which the associations between perfectionism and CDMD may be explained, thereby contributing to more targeted and effective support in career decision-making for students characterized by elevated perfectionism. This study makes the following hypothesis based on the analysis above:

*H3a*: Psychological resilience acts as a partial mediator between positive perfectionism and CDMD.

*H3b*: Psychological resilience acts as a partial mediator between negative perfectionism and CDMD.

### 2.5 The chain mediating role of core self-evaluations and psychological resilience

Previous research has shown that psychological resilience and core self-evaluations are positively correlated (Li et al., 2020; Zeb et al., 2025). From the perspective of CBT and SCCT, core self-evaluations are closer to students’ relatively stable general self-belief system, including competence, self-worth, emotional stability, and control, whereas psychological resilience reflects how these self-beliefs are mobilized when students face stress, uncertainty, and setbacks in career decision-making. Core self-evaluations represent fundamental evaluations of one’s competence and worth and have been linked to stress appraisal and coping processes (Kammeyer-Mueller et al., 2009). Self-evaluations are also strongly linked to career hesitation and decision-making challenges in contexts involving career decision-making (Udayar et al., 2020). Therefore, students with higher core self-evaluations could be more inclined to think they can handle career-related difficulties, maintain emotional stability, and retain a sense of control under uncertainty, which provides a cognitive basis for psychological resilience. Higher core self-evaluations are linked to stronger psychological resilience, according to empirical research (Hong and Wang, 2024). However, among college students, resilience is strongly associated with CDMD and career adaptability (Pang et al., 2021; Wan et al., 2023). Thus, core self-evaluations are positioned before psychological resilience in the proposed chain mediation model, while both are treated as closely related psychological resources rather than as strictly separated processes. Because perfectionism may still be directly associated with CDMD through cognitive-evaluative tendencies such as fear of mistakes, self-doubt, rigid standards, and planning tendencies, the present study expects partial rather than full mediation.

*H4a*: Core self-evaluations and psychological resilience partially and sequentially mediate the association between positive perfectionism and CDMD.

*H4b*: Core self-evaluations and psychological resilience partially and sequentially mediate the association between negative perfectionism and CDMD.

**Figure 1:**
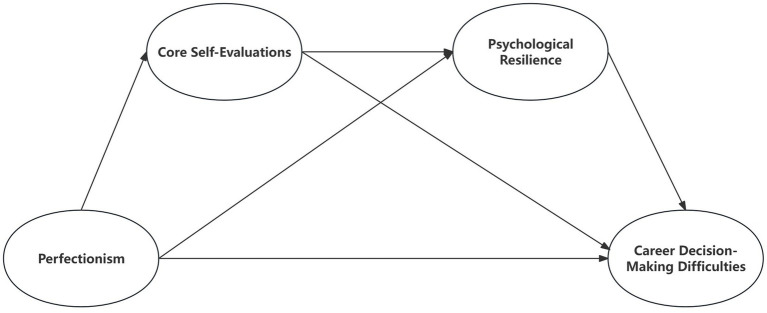
Research model.

The research model for this study is depicted in Figure 1.

## 3 Research methods

### 3.1 Sample and data collection

The Dongguan Polytechnic Ethics Review Committee approved this study. The study was conducted among college students from five higher education institutions located in Guangdong and Guangxi provinces in China. These institutions included four public institutions and one private college, covering both undergraduate institutions and a higher vocational college. The sampling frame consisted of students from accessible class groups in the cooperating institutions. Data were collected through Wenjuanxing during May and June 2025 with the assistance of academic staff from the collaborating institutions. The questionnaire link was distributed through class groups, and students participated voluntarily after reading the study information and providing informed consent. The study procedures followed the ethical principles of the Declaration of Helsinki, and privacy protection measures were emphasized. Because participants were recruited through accessible class groups rather than probability sampling, this study adopted a convenience sampling procedure, and the representativeness of the sample should be interpreted with caution.

Sample size adequacy was evaluated using a Monte Carlo power analysis for indirect effects following Schoemann et al. (2017). A target power of 0.80 was adopted as the conventional threshold for adequate statistical power (Cohen, 1988). Separate analyses were conducted for the positive and negative perfectionism models, with core self-evaluations and psychological resilience specified as two serial mediators. Using standardized coefficients, 5,000 replications, 10,000 Monte Carlo draws per replication, *α* = 0.05, and 95% confidence intervals, the results showed that when the sample size was set at 210, the power values for the three indirect effects were 0.89, 0.82, and 0.91 in the positive perfectionism model, and 0.97, 0.80, and 0.96 in the negative perfectionism model. Thus, 210 valid responses met the conventional power criterion, and the final valid sample of 1,000 participants exceeded this requirement.

A total of 1,050 questionnaires were collected. After data screening, 1,000 valid responses were retained, yielding an effective response rate of 95.24%. Invalid questionnaires were identified based on incomplete responses and response-quality indicators. Specifically, 21 questionnaires were excluded because the completion rate was below 80%, 13 were excluded because of highly repetitive and careless response patterns, and 16 were excluded because of multiple response-quality concerns, such as repetitive, extreme, or inconsistent response patterns identified during manual screening. To avoid excluding valid respondents solely on the basis of response similarity, questionnaires with more than 80% identical or extreme responses were not automatically removed; instead, they were further inspected together with the overall response pattern. Cases were excluded only when the response pattern suggested insufficient engagement. Table 1 presents the demographic characteristics of the participants and the results of preliminary difference tests for CDMD across gender, age, grade level, and educational background. No significant differences in CDMD were found across these demographic groups.

**Table 1:** Demographic information of the participants.

Demographic characteristics	Category	Frequency	Percentage (%)	*F*/*t*	*p*
Gender	Male	345	34.50	−0.911	0.362
	Female	655	65.50		
Age	Under 18 years old	4	0.40	0.315	0.814
	18~20	363	36.30		
	20~22	411	41.10		
	Over 22 years old	222	22.20		
Grade level	Freshmen	208	20.80	0.910	0.436
	Sophomores	268	26.80		
	Juniors	320	32.00		
	Seniors	204	20.40		
Educational background	Humanities	209	20.90	2.461	0.086
	Social sciences	569	56.90		
	Natural sciences	222	22.20		

### 3.2 Measurement tools

The Chinese version of the FMPS, originally developed by Frost et al. (1990) and translated and revised by Zi and Zhou (2006), was used to measure perfectionism in this study. The scale contains 27 items covering five dimensions: organization, parental expectations, personal standards, concern over mistakes, and doubts about actions. Consistent with previous applications of the Chinese FMPS, the organization dimension was used to represent positive perfectionism, whereas parental expectations, personal standards, concern over mistakes, and doubts about actions were combined to represent negative perfectionism (Hu et al., 2023). In this measurement framework, positive perfectionism mainly reflects orderliness, planning, and preference for structure, while negative perfectionism reflects concerns about mistakes, self-doubt in action, parental expectations, and high personal standards. A each question was graded using a 5-point Likert scale; higher scores indicated higher levels of the associated perfectionism characteristic. Prior studies with Chinese college student samples have supported the reliability and validity of this scale (Liu and Sun, 2026). Cronbach’s alpha values for positive and negative perfectionism in this study were 0.843 and 0.938, respectively. Two subscales showed satisfactory model fit according to confirmatory factor analysis: *χ*^2^/d*f* = 2.556, RMSEA = 0.039, TLI = 0.973, CFI = 0.975, GFI = 0.947, AGFI = 0.936, IFI = 0.973 for negative perfectionism and *χ*^2^/d*f* = 2.791, RMSEA = 0.042, TLI = 0.987, CFI = 0.992, GFI = 0.992, AGFI = 0.980, IFI = 0.992 for positive perfectionism.

The Core Self-Evaluations Scale was used to measure core self-evaluations, according to Judge et al. (2003) and Sun and Jiang (2017). Ten questions (such as “I am certain that I can manage the majority of chores”) are judged using a 5-point Likert scale. Self-perceptions that are more positive are correlated with higher scores. The measure has demonstrated strong validity and reliability in Chinese college student populations (Qian et al., 2023). In this investigation, Cronbach’s *α* was 0.921, and the confirmatory factor analysis (*χ*^2^/d*f* = 1.880; RMSEA = 0.030, TLI = 0.992, CFI = 0.994, GFI = 0.988, AGFI = 0.981, IFI = 0.994) produced a satisfactory fit.

The Connor-Davidson Resilience Scale’s Chinese version was used to evaluate psychological resilience (Connor and Davidson, 2003; Yu and Zhang, 2007). In three dimensions, self-strength, optimism, and perseverance, the measure comprises 25 items (e.g., “I am capable of managing uncomfortable emotions like fear, rage, and grief”). A 5-point Likert scale is used to grade responses (1 being “never” and 5 being “always”). Higher overall scores indicate stronger resilience. In samples of Chinese college students, this instrument has shown strong validity and reliability (Cai et al., 2023). Confirmatory factor analysis revealed a decent model fit (*χ*^2^/d*f* = 1.374, RMSEA = 0.019, TLI = 0. 993, CFI = 0.993, GFI = 0.971, AGFI = 0.966, IFI = 0.993), and the scale in this study demonstrated good internal consistency (Cronbach’s *α* = 0.965).

CDMD were measured using the Chinese Career Decision-Making Difficulties Scale developed by Du and Long (2006) and used in recent research by Zhou A. B. et al. (2024). The scale contains 16 items covering four dimensions: career information exploration, career self-exploration, career planning exploration, and career goal exploration. The items are positively worded to reflect career exploration and planning behaviors, such as “I pay close attention to career-related information” and “I have clear long-term career goals.” Because the present study focused on CDMD, all items were reverse-scored before calculating the total score. After reverse scoring, higher scores indicated lower levels of career exploration and planning, that is, greater CDMD. Each item was rated on a 5-point Likert scale. A satisfactory model fit was demonstrated by Cronbach’s *α* = 0.941, the scale’s internal consistency, and the confirmatory factor analysis (*χ*^2^/d*f* = 1.522; RMSEA = 0.023, TLI = 0. 992, CFI = 0.993, GFI = 0.981, AGFI = 0.975, IFI = 0.992).

### 3.3 Statistical analysis

For this study, data analysis was done using AMOS 29.0 and SPSS 24.0. First, descriptive statistics and normality tests were performed on each variable. The data were deemed to be relatively regularly distributed when the absolute values of skewness and kurtosis were less than two and seven, respectively (Kim, 2013). After that, Pearson correlation analysis was used to look at the connections between positive perfectionism, negative perfectionism, core self-evaluations, psychological resilience, and CDMD. Second, Confirmatory factor analysis was performed using AMOS 29.0 to assess the measurement model’s fit. Scale reliability and convergent validity were assessed using Cronbach’s *α*, composite reliability (CR), and average variance extracted (AVE). The Fornell-Larcker criterion and the heterotrait-monotrait ratio (HTMT) were used to evaluate discriminant validity. To test for common method bias, this study initially conducted a preliminary assessment using Harman’s single-factor test; on this basis, a supplementary test was further carried out using the common latent factor method (CLF). In addition, before testing the mediation models, this study conducted preliminary analyses of the relationships between gender, age, grade level, academic discipline, and CDMD. Since the results showed that these demographic variables were not significantly associated with CDMD, they were left out of the control variables in the mediation models that followed in order to preserve consistency between the model specification and the research hypotheses and to avoid the introduction of unnecessary covariates. Finally, two PROCESS Model 6 analyses were conducted to examine the theory-informed serial indirect associations in the positive perfectionism model and the negative perfectionism model, with core self-evaluations and psychological resilience specified as sequential mediators. Given the cross-sectional design, these analyses were interpreted as tests of statistical mediation rather than causal mediation, and the specified ordering of variables was treated as theory-informed rather than temporal. Indirect effects were estimated using 5,000 bootstrap samples, and their statistical significance was determined by whether the 95% confidence interval contained 0.

## 4 Results

### 4.1 Common method Bias test

First, Harman’s single-factor test was used to perform a preliminary assessment of common technique bias. Five factors with eigenvalues greater than 1 were found in the results. The first factor explained 33.169% of the total variance, which was less than the 40% reference level (Podsakoff and Organ, 1986). A similar latent factor technique was also employed as an additional test. The findings demonstrated that, when compared to the baseline measurement model, adding a CLF did not significantly enhance model fit (Δ*χ*^2^ = 2.023, Δd*f* = 1, *p* > 0.05). These data imply that there was insufficient proof that the results were significantly impacted by common technique bias.

### 4.2 Descriptive statistics and correlation analysis

Each variable’s skewness and kurtosis satisfy requirement (Kim, 2013), as Table 2 illustrates, so Pearson correlation analysis was adopted. According to Cohen (1988) guidelines, Small, medium, and high effects are denoted by absolute correlation values of roughly 0.10, 0.30, and 0.50, respectively. The directions of the correlations were consistent with the hypotheses: positive perfectionism, core self-evaluations, and psychological resilience were negatively associated with CDMD, whereas negative perfectionism was positively associated with CDMD. In terms of effect size, the correlations between CDMD and positive perfectionism, negative perfectionism, core self-evaluations, and psychological resilience all fell within the medium range, with the associations involving positive perfectionism and core self-evaluations approaching the large-effect threshold. The correlations between positive perfectionism and the two psychological resources reached the large-effect range, whereas the correlations between negative perfectionism and the two psychological resources remained in the medium range. Positive and negative perfectionism also showed a medium negative association, indicating that the two perfectionism dimensions were related but not redundant. The correlation between core self-evaluations and psychological resilience reached the large-effect range, which was consistent with their conceptual relatedness as psychological resources. Their empirical distinctiveness was further evaluated in the subsequent measurement model using the Fornell-Larcker criterion and HTMT ratios.

**Table 2:** Data description and correlation analysis between PP, NP, CSE, PR, and CDMD.

Variables	*M* ± SD	SK	Kur	PP	NP	CDMD	PR	CSE
PP	19.760 ± 4.382	−0.812	−0.453	1				
NP	65.461 ± 15.461	−0.194	−1.637	−0.366***	1			
CDMD	42.354 ± 10.668	1.039	−0.269	−0.497***	0.434***	1		
PR	74.060 ± 18.641	−0.080	−1.616	0.519***	−0.455***	−0.454***	1	
CSE	29.334 ± 7.607	0.100	−1.473	0.514***	−0.476***	−0.478***	0.664***	1

***p < 0.001; PP, positive perfectionism; NP, negative perfectionism; CSE, core self-evaluations; PR, psychological resilience; CDMD, career decision-making difficulties.

### 4.3 Confirmatory factor analysis

On the basis of the separate CFAs conducted for each scale, this study further tested a full measurement model including five latent variables: positive perfectionism, negative perfectionism, core self-evaluations, psychological resilience, and CDMD. The whole measurement model demonstrated a good fit, according to the results: *χ*^2^/d*f* = 1.364, RMSEA = 0.019, TLI = 0.976, CFI = 0.976, GFI = 0.908, AGFI = 0.902, and IFI = 0.977. These results suggest that the measurement structure of the five latent variables was consistent with the data.

### 4.4 Reliability and validity

Before conducting the mediation analysis, this study used SPSS to assess each indicator’s reliability and validity. According to the findings, every scale had Cronbach’s alpha values more than 0.7, which denotes strong reliability (Brown, 2002). CR also demonstrated high reliability. The scales appear to be genuine and able to measure the constructs because the AVE values were greater than 0.5 (Henseler et al., 2015). The results of the validity and reliability tests for each variable are shown in Table 3.

**Table 3:** Reliability and validity test results.

Variables	Cronbach’s alpha	CR	AVE
PP	0.843	0.884	0.560
NP	0.938	0.945	0.534
CDMD	0.941	0.948	0.531
PR	0.965	0.967	0.543
CSE	0.921	0.933	0.584

PP, positive perfectionism; NP, negative perfectionism; CSE, core self-evaluations; PR, psychological resilience; CDMD, career decision-making difficulties.

As Table 4 shows, the Fornell-Larcker criterion was met since each construct’s square root of the AVE was higher than its correlations with other constructs. In addition, all HTMT values were below 0.85, as shown in Table 5, providing further support for acceptable discriminant validity among the latent variables. Although core self-evaluations and psychological resilience were relatively highly correlated, their HTMT value remained below the recommended threshold, indicating sufficient construct distinctiveness in the present study.

**Table 4:** Fornell-Larcker criterion.

Variables	PR	CSE	NP	PP	CDMD
PR	**0.737**				
CSE	0.665	**0.764**			
NP	−0.449	−0.475	**0.731**		
PP	0.531	0.525	−0.369	**0.748**	
CDMD	−0.456	−0.48	0.432	−0.501	**0.729**

On the table’s diagonal, the square root of the AVE values are shown in bold; PP, positive perfectionism; NP, negative perfectionism; CSE, core self-evaluations; PR, psychological resilience; CDMD, career decision-making difficulties.

**Table 5:** Heterotrait-monotrait criterion.

Variables	PR	CSE	NP	PP	CDMD
PR					
CSE	0.704				
NP	0.469	0.509			
PP	0.577	0.585	0.409		
CDMD	0.477	0.513	0.457	0.558	

PP, positive perfectionism; NP, negative perfectionism; CSE, core self-evaluations; PR, psychological resilience; CDMD, career decision-making difficulties.

### 4.5 Serial indirect association analysis

**Figure 2:**
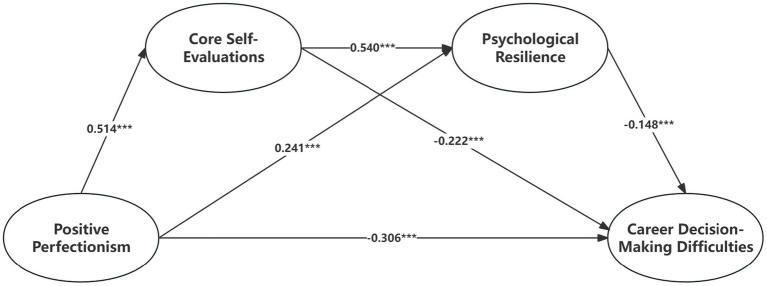
Path coefficients of the direct effects of PP, CSE, PR, and CDMD; ***p < 0.001.

**Figure 3:**
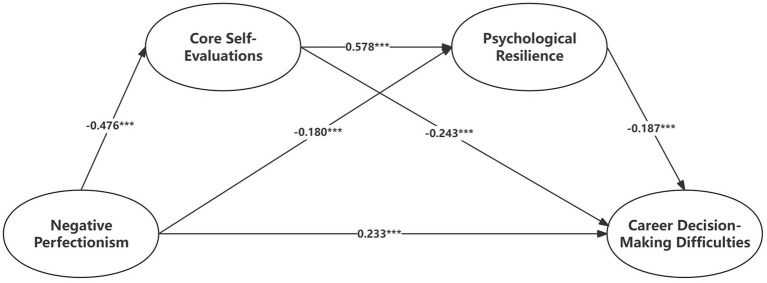
Path coefficients of the direct effects of NP, CSE, PR, and CDMD; ***p < 0.001.

Tables 6, 7 present the path results for the two chain-mediation models (Figures 2, 3). In the positive perfectionism model, positive perfectionism was substantially and inversely connected with CDMD and considerably and favorably correlated with psychological resilience and core self-evaluations. Core self-evaluations were significantly and positively associated with psychological resilience, and both were significantly and negatively associated with CDMD. In the negative perfectionism model, Negative perfectionism was positively and strongly correlated with CDMD and negatively and significantly correlated with psychological resilience and core self-evaluations. Core self-evaluations and psychological resilience were both negatively associated with CDMD, and core self-evaluations were positively associated with psychological resilience. The above results show that the direct path directions in both sets of models are consistent with the research hypotheses.

**Table 6:** Chain mediation model regression analysis of PP, CSE, PR, and CDMD (*N* = 1,000).

Outcome variable	Predictive variable	*R* ^2^	*F*	*β*	SE	*t*	95% CI
Equation 1							
CSE	PP	0.264	358.531***	0.514	0.027	18.935***	[0.461, 0.567]
Equation 2							
PR	PP	0.483	466.568***	0.241	0.027	9.080***	[0.189, 0.293]
	CSE			0.540	0.027	20.349***	[0.488, 0.592]
Equation 3							
CDMD	PP	0.326	160.308***	−0.306	0.032	−9.700***	[−0.368, −0.244]
	CSE			−0.222	0.036	−6.159***	[−0.293, −0.151]
	PR			−0.148	0.036	−4.085***	[−0.219, −0.077]

***p < 0.001; PP, positive perfectionism; CSE, core self-evaluations; PR, psychological resilience; CDMD, career decision-making difficulties.

**Table 7:** Chain mediation model regression analysis of NP, CSE, PR, and CDMD (*N* = 1,000).

Outcome variable	Predictive variable	*R* ^2^	*F*	*β*	SE	*t*	95% CI
Equation 1							
CSE	NP	0.227	292.734***	−0.476	0.028	−17.109***	[−0.531, −0.422]
Equation 2							
PR	NP	0.466	434.627***	−0.180	0.026	−6.385***	[−0.232, −0.128]
	CSE			0.578	0.026	21.964***	[0.527, 0.630]
Equation 3							
CDMD	NP	0.302	143.702***	0.233	0.031	7.571***	[0.173, 0.294]
	CSE			−0.243	0.037	−6.619***	[−0.315, −0.171]
	PR			−0.187	0.036	−5.161***	[−0.258, −0.116]

***p < 0.001; NP, negative perfectionism; CSE, core self-evaluations; PR, psychological resilience; CDMD, career decision-making difficulties.

Table 8 further shows that the total indirect associations in both the positive perfectionism model and the negative perfectionism model were statistically significant, and the bootstrap 95% confidence intervals for each separate indirect path and the serial indirect path did not include zero. The results showed that, in the positive perfectionism model, the separate path through core self-evaluations, the separate path through psychological resilience, and the serial path through core self-evaluations and psychological resilience were all statistically significant. Further comparisons of the indirect associations showed that, in the positive perfectionism model, the path through core self-evaluations was significantly stronger than the path through psychological resilience, C1 = −0.079, 95% CI [−0.129, −0.029]. Additionally, the path via core self-evaluations was substantially stronger than the serial path via psychological resilience and core self-evaluations (C2 = −0.073, 95% CI [−0.126, −0.021]). The psychological resilience path and the serial path did not significantly differ from one another (C3 = 0.005, 95% CI [−0.006, 0.020]). These results suggest that the statistical indirect association between positive perfectionism and CDMD was mainly reflected at the level of self-evaluations.

**Table 8:** Mediation effect analysis of PP, NP, CSE, PR, and CDMD (*N* = 1,000).

Effect	*β*	Boot SE	Boot 95% CI	Effect proportion
Model 1				
Total effect	−0.497	0.027	[−0.551, −0.443]	100.000%
Direct effect	−0.306	0.032	[−0.368, −0.244]	61.569%
Total indirect effect	−0.191	0.019	[−0.230, −0.155]	38.431%
Ind1_Model 1_	−0.114	0.020	[−0.153, −0.077]	59.686%
Ind2_Model 1_	−0.036	0.009	[−0.054, −0.018]	18.848%
Ind3_Model 1_	−0.041	0.011	[−0.064, −0.020]	21.466%
Model 2				
Total effect	0.434	0.029	[0.378, 0.490]	100.000%
Direct effect	0.233	0.031	[0.173, 0.294]	53.687%
Total indirect effect	0.201	0.019	[0.165, 0.239]	46.313%
Ind1_Model 2_	0.116	0.018	[0.082, 0.152]	57.711%
Ind2_Model 2_	0.034	0.009	[0.018, 0.052]	16.915%
Ind3_Model 2_	0.051	0.011	[0.031, 0.074]	25.373%

***p < 0.001. Ind1_Model 1_, positive perfectionism → core self-evaluations → career decision-making difficulties; Ind2_Model 1_, positive perfectionism → psychological resilience → career decision-making difficulties; Ind3_Model 1_, positive perfectionism → core self-evaluations → psychological resilience → career decision-making difficulties; Ind1_Model 2_, negative perfectionism → core self-evaluations → career decision-making difficulties; Ind2_Model 2_, negative perfectionism → psychological resilience → career decision-making difficulties; Ind3_Model 2_, negative perfectionism → core self-evaluations → psychological resilience → career decision-making difficulties.

In the negative perfectionism model, the three indirect paths were also statistically significant. Unlike the positive perfectionism model, the proportions of the three indirect paths in the negative perfectionism model were more similar, suggesting that the statistical indirect association between negative perfectionism and CDMD involved both core self-evaluations and psychological resilience, rather than being concentrated in a single psychological resource path. The path through core self-evaluations was significantly stronger than the path through psychological resilience, C1 = 0.082, 95% CI [0.039, 0.128]. The path through core self-evaluations was also significantly stronger than the serial path through core self-evaluations and psychological resilience, C2 = 0.064, 95% CI [0.019, 0.113]. The serial path through core self-evaluations and psychological resilience was significantly stronger than the path through psychological resilience, C3 = −0.018, 95% CI [−0.037, −0.003]. These results indicate that, in the statistical indirect association between negative perfectionism and CDMD, core self-evaluations represented a more prominent psychological resource component. Although psychological resilience showed an independent indirect association, it was more closely involved in the serial path together with core self-evaluations.

## 5 Discussion

By differentiating between positive and negative perfectionism, this study looked at the relationship between college students’ perfectionism and CDMD. Consistent with previous findings (Ji et al., 2024; Si and Lee, 2022), positive perfectionism was negatively associated with CDMD, whereas negative perfectionism was positively associated with CDMD. From a CBT perspective, negative perfectionism reflects maladaptive cognitive-evaluative tendencies, such as strict guidelines, extreme worry about errors, uncertainty regarding deeds, and self-critical assessment (Beck et al., 2024). These tendencies may be associated with greater hesitation, self-doubt, and difficulty when students evaluate career options under uncertainty (Muliasari et al., 2019). In contrast, positive perfectionism in this study was represented by the organization dimension of the Chinese FMPS, which mainly reflects orderliness, planning, and structured goal pursuit (Cao et al., 2024). Its negative association with CDMD suggests that students with stronger organization-related perfectionistic tendencies may report more systematic information collection, clearer comparison of alternatives, and more organized career planning. These findings indicate that different components of perfectionism may show distinct associations with CDMD, while also highlighting the need to interpret positive perfectionism within the measurement framework used in this study.

Core self-evaluations, according to this study, partially accounted for the connection between the two positive perfectionism, negative perfectionism, and CDMD. This outcome is consistent with the conclusions of Fearn et al. (2022) and Shen et al. (2021). Specifically, for individuals with positive perfectionism, they typically demonstrate higher self-efficacy and self-esteem, believing that “I can handle most challenges.” This confidence may be associated with greater decisiveness in career decisions and may be linked to better tolerance of pressure and uncertainty, relating to fewer CDMD (Pignault et al., 2023). In contrast, individuals with negative perfectionism often have lower core self-evaluations, expressed as “I often feel inadequate” or “I cannot handle certain challenges.” These negative self-perceptions may be associated with increased anxiety and self-doubt when making career decisions, which may be linked to decision avoidance and difficulties (Shi, 2023). At the same time, previous career research has also shown that prolonged career indecision and repeated decision setbacks may be associated with lower self-efficacy, weaker self-worth, and more negative self-appraisals (Pignault et al., 2023; Udayar et al., 2020). This indicates that the association between core self-evaluations and CDMD should not be understood as a one-way process only. Instead, students’ self-evaluations and their career decision-making experiences may be dynamically related during career development. To investigate the temporal ordering and potential reciprocal relationships between these variables, more longitudinal or cross-lagged research is required.

Psychological resilience was found to partially account for the link between both forms of perfectionism, positive and negative, and CDMD, in line with earlier studies by Islim and Ahmad (2025) and Pang et al. (2021). On one hand, organization-based positive perfectionism tendencies could be connected to higher psychological resilience in college students, reflected in stronger stress tolerance and more positive coping, which may be related to lower sensitivity to the decision-making pressure that may result from high standards, relate to stronger career adaptability, and thus be associated with a lower likelihood of encountering CDMD (Alkal, 2025). In contrast, negative perfectionism tends to be connected to a lower psychological resilience (e.g., increasing self-doubt and triggering avoidance behaviors) (Fearn et al., 2022; Mansy et al., 2024), which may be linked to greater susceptibility during the decision-making process, making choosing a career more challenging. On the other hand, from the perspective of SCCT (Lent et al., 1994), these mediating mechanisms reflect the associations of individual resources with the cognitive-behavioral path: Positive perfectionists, by setting achievable high standards, may report higher career self-efficacy, thereby relating to higher psychological resilience for coping with career uncertainty (Kruger et al., 2023). In contrast, negative perfectionism, with its rigid goal-setting and fear of failure, may be associated with lower self-efficacy beliefs, relating to lower psychological resilience reserves (Kruger et al., 2023). However, resilience is not only a stable personal resource; it may also vary with students’ accumulated experiences of success, frustration, and uncertainty in career exploration. For students who repeatedly encounter obstacles in career decision-making, their perceived coping capacity may become weaker over time, which may further intensify hesitation and avoidance. This possibility is consistent with studies showing close links between CDMD, psychological resilience, and career adaptability (He et al., 2024; Pang et al., 2021).

Core self-evaluations and psychological resilience were also successively linked to the connection between perfectionism and CDMD, indicating an important chain mediation route. This pattern is in line with the conclusions of Hong and Wang (2024) and conceptually aligned with CBT and SCCT (Beck et al., 2024; Lent et al., 1994). The proposed sequence is theoretically reasonable because core self-evaluations reflect students’ general self-beliefs regarding competence, worth, and control, whereas psychological resilience reflects their adaptive capacity when facing stress, setbacks, and uncertainty. When making professional decisions, students’ fundamental assessments of their skills and self-worth may provide a cognitive basis for maintaining resilience when they face unclear career information, employment pressure, and possible decision setbacks. However, this pathway should not be interpreted as a fixed temporal process. SCCT emphasizes the interplay among personal beliefs, behaviors, and contextual experiences (Bandura, 1986; Lent et al., 1994), and research suggests that general self-beliefs and resilience-related resources may influence each other over time (Kammeyer-Mueller et al., 2009; Wu and Griffin, 2012). Therefore, the ordering of core self-evaluations and psychological resilience in this study represents a theory-informed analytical arrangement rather than a confirmed developmental sequence. This finding suggests that career counseling in universities should focus on students’ self-understanding and resilience-related resources, especially for those with stronger negative perfectionistic concerns.

## 6 Implications and limitations

### 6.1 Theoretical implications

First, this study extends the application of CBT to the connection between perfectionism and CDMD by examining the roles of core self-evaluations and psychological resilience. By distinguishing organization-based positive perfectionism tendencies from negative perfectionistic concerns, the study provides a more differentiated understanding of perfectionism in career decision-making contexts. Second, the study suggests that psychological resilience is a relevant psychological resource in career decision-making, thereby extending the application of SCCT to the interpretation of students’ career-related difficulties. Finally, the study offers preliminary empirical support for an integrated model of CDMD among college students, emphasizing the interrelated associations among perfectionism, core self-evaluations, and psychological resilience.

### 6.2 Practical implications

For college students, this study suggests that career decision-making support should help them identify negative perfectionistic tendencies, manage excessive fear of mistakes, and distinguish structured goal pursuit from rigid self-demand. Maintaining order, planning, and preparation may support career exploration, whereas excessive self-criticism may increase hesitation and avoidance. Students may also benefit from developing more flexible standards, accepting uncertainty, and maintaining confidence in their abilities during career preparation in their abilities during career preparation.

For career consultants and psychologists, the findings provide empirical reference for counseling strategies focused on perfectionistic concerns, self-evaluations, and resilience-related resources. Counselors can guide students to reflect on unrealistic standards, develop more balanced self-awareness, clarify career interests and abilities, and set realistic career goals. Career guidance can also include structured support to help students cope with stress and uncertainty during career preparation.

For families and the broader social environment, the findings suggest the importance of providing realistic feedback, emotional acceptance, and autonomy support. Families should avoid reinforcing rigid standards such as “must choose the best path” or “must avoid failure.” Universities and social organizations can also provide diverse career resources, psychological counseling services, and career development activities to support more informed and flexible career development.

### 6.3 Limitations and future research directions

Even though this research provides helpful factual support for understanding the associations among perfectionism, core self-evaluations, psychological resilience, and CDMD, there are a few restrictions to take into account.

First, the interpretation of the chain mediation model is constrained by the cross-sectional design. The significant indirect associations found in this study should not be interpreted as proof of temporal change or causal effects among the variables. In particular, the findings cannot determine whether perfectionism precedes later changes in core self-evaluations or psychological resilience, nor can they verify the temporal ordering between core self-evaluations and psychological resilience. Although the proposed order was based on the theoretical distinction between general self-beliefs and resilience-related resources, SCCT emphasizes that personal beliefs and career-related experiences may be dynamically interrelated, and longitudinal designs are needed to examine possible reciprocal associations among these variables. Future longitudinal, multi-wave, or cross-lagged studies could examine the temporal ordering and potential reciprocal relationships.

Second, although this study followed previous applications of the Chinese FMPS in distinguishing positive and negative perfectionism, this classification may not fully capture the broader multidimensional structure of perfectionism. In the present study, positive perfectionism was represented by organization, which mainly reflects orderliness and planning. Future research could examine the five FMPS dimensions separately or distinguish perfectionistic strivings, perfectionistic concerns, and organization to provide a more fine-grained understanding of perfectionism in career decision-making contexts.

Third, the self-report measures used in this study were gathered from a single source. Shared method variance and social desirability bias cannot be completely ruled out, despite Harman’s single-factor test and the CLF methodology suggesting that common method bias was not a significant worry. These issues should be considered when interpreting the strength of the observed associations. To improve internal validity, future research could include time-lagged designs, behavioral markers, interview data, or multi-source data.

Finally, Chinese college students made up the sample, which would restrict how broadly the results can be applied. Both perfectionism and CDMD are shaped by sociocultural contexts, such as educational competition, family expectations, and labor market conditions. Future studies could examine whether the observed associations remain stable across different cultural, educational, and occupational contexts.

## 7 Conclusion

Grounded in CBT and SCCT, this investigation looked at the relationships between perfectionism, core self-evaluations, psychological resilience, and CDMD among college students. The findings suggest that positive and negative perfectionism are related to CDMD in different directions. Core self-evaluations and psychological resilience were also involved in these associations, both separately and sequentially. These findings offer initial empirical evidence in favor of understanding CDMD from the perspective of perfectionistic tendencies and psychological resources. Future longitudinal and multi-source studies are needed to further clarify the temporal ordering and generalizability of these associations.

## Data Availability

The raw data supporting the conclusions of this article will be made available by the authors, without undue reservation.
